# Supplementary material for: MetaRibo-Seq measures translation in microbiomes
Source: Nat Commun. 2020 Jun 29;11:3268. doi: 10.1038/s41467-020-17081-z (PMC7324362; doi:10.1038/s41467-020-17081-z)
Supplement: Supplementary file 10 — Supplementary Data 7 [file 41467_2020_17081_MOESM10_ESM.zip › File2/Confidence_High_Taxonomy/135834_out.krona.html]

Javascript must be enabled to view this page.

members
magnitude
magnitudeUnassigned
count
unassigned
taxon
rank

135834\_out

97

2
superkingdom
97

phylum
97
1239

186801
97
class

186802
order
97

186803
97
family

841
97
genus

species

SRS011084\_contig\_number\_37329SRS011405\_contig\_number\_17539SRS012849\_contig\_number\_23168SRS013951\_contig\_number\_33533SRS014235\_contig\_number\_29814SRS014412\_contig\_number\_21131SRS014923\_contig\_number\_36411SRS014948\_contig\_number\_contig-100\_29176.29177SRS015065\_contig\_number\_contig-100\_802.200112SRS015264\_contig\_number\_6498SRS015486\_contig\_number\_contig-100\_5531.5532SRS015578\_contig\_number\_37202SRS015663\_contig\_number\_12330SRS015663\_contig\_number\_34082SRS015854\_contig\_number\_2929SRS015890\_contig\_number\_16526SRS016018\_contig\_number\_contig-100\_26993.26993SRS016095\_contig\_number\_12946SRS018575\_contig\_number\_contig-100\_11423.11423SRS018984\_contig\_number\_22428SRS019068\_contig\_number\_67047SRS019068\_contig\_number\_99226SRS019161\_contig\_number\_39842SRS019381\_contig\_number\_17140SRS019397\_contig\_number\_13378SRS019445\_contig\_number\_36280SRS022609\_contig\_number\_2979SRS023346\_contig\_number\_6918SRS024388\_contig\_number\_10238SRS024492\_contig\_number\_9796SRS042628\_contig\_number\_35459SRS045645\_contig\_number\_5304SRS046369\_contig\_number\_contig-100\_18148.18149SRS046502\_contig\_number\_contig-100\_13601.13602SRS048164\_contig\_number\_25420SRS049446\_contig\_number\_contig-100\_25551.25552SRS049959\_contig\_number\_45228SRS050026\_contig\_number\_contig-100\_8578.44917SRS050925\_contig\_number\_contig-100\_2971.83462SRS050998\_contig\_number\_12712SRS051031\_contig\_number\_14173SRS052027\_contig\_number\_5180SRS052697\_contig\_number\_contig-100\_23830.116255SRS053398\_contig\_number\_13082SRS054956\_contig\_number\_contig-100\_24739.24739SRS055017\_contig\_number\_contig-100\_40219.40220SRS056537\_contig\_number\_56236SRS058070\_contig\_number\_29524SRS058770\_contig\_number\_26157SRS063040\_contig\_number\_42057SRS063518\_contig\_number\_contig-100\_7719.128337SRS064645\_contig\_number\_contig-100\_368.368SRS075021\_contig\_number\_contig-100\_1209.54457SRS076804\_contig\_number\_contig-100\_8058.96495SRS078419\_contig\_number\_contig-100\_13505.48003SRS097889\_contig\_number\_36277SRS097958\_contig\_number\_contig-100\_28669.66825SRS098827\_contig\_number\_10012SRS098881\_contig\_number\_contig-100\_666.147988SRS100021\_contig\_number\_contig-100\_7272.7272SRS103987\_contig\_number\_3824SRS104693\_contig\_number\_12191SRS105153\_contig\_number\_38292SRS1054716\_contig\_number\_1079SRS1055022\_contig\_number\_contig-100\_6935.105366SRS1055056\_contig\_number\_contig-100\_1036.1037SRS140492\_contig\_number\_contig-100\_3362.161775SRS142503\_contig\_number\_contig-100\_3458.223203SRS142890\_contig\_number\_19959SRS143085\_contig\_number\_contig-100\_4696.4697SRS143417\_contig\_number\_33321SRS143722\_contig\_number\_15444SRS144135\_contig\_number\_20882SRS144297\_contig\_number\_12218SRS144362\_contig\_number\_31325SRS144506\_contig\_number\_56277SRS144714\_contig\_number\_26550SRS146764\_contig\_number\_33106SRS147766\_contig\_number\_21206SRS147977\_contig\_number\_13181SRS148091\_contig\_number\_10629SRS148159\_contig\_number\_52994SRS148253\_contig\_number\_14142SRS148424\_contig\_number\_29606SRS149784\_contig\_number\_contig-100\_14818.100954SRS893259\_contig\_number\_4957SRS893270\_contig\_number\_contig-100\_2261.2262SRS893288\_contig\_number\_contig-100\_117.29075SRS893300\_contig\_number\_contig-100\_15931.15932SRS893327\_contig\_number\_contig-100\_247.72129SRS893342\_contig\_number\_8383
91
360807

species

SRS016335\_contig\_number\_44898SRS017433\_contig\_number\_15429SRS056519\_contig\_number\_10959SRS097920\_contig\_number\_32135SRS143148\_contig\_number\_26187SRS148784\_contig\_number\_29239
6
1263105
